# Profiling networks of distinct immune-cells in tumors

**DOI:** 10.1186/s12859-016-1141-3

**Published:** 2016-07-04

**Authors:** Trevor Clancy, Eivind Hovig

**Affiliations:** Department of Tumor Biology, Institute for Cancer Research, The Norwegian Radium Hospital, Oslo University Hospital, Oslo, Norway; Department of Cancer Immunology, Institute for Cancer Research, The Norwegian Radium Hospital, Oslo University Hospital, Oslo, Norway; Biomedical Research Group, Department of Informatics, Faculty of Mathematics and Natural Sciences, University of Oslo, Oslo, Norway; Institute of Cancer Genetics and Informatics, The Norwegian Radium Hospital, Oslo University Hospital, Oslo, Norway

**Keywords:** Immune profiling, Cancer, Transcriptomics, Immune informatics, Personalized medicine, Protein interaction networks, Immune-cell infiltration

## Abstract

**Background:**

It is now clearly evident that cancer outcome and response to therapy is guided by diverse immune-cell activity in tumors. Presently, a key challenge is to comprehensively identify networks of distinct immune-cell signatures present in complex tissue, at higher-resolution and at various stages of differentiation, activation or function. This is particularly so for closely related immune-cells with diminutive, yet critical, differences.

**Results:**

To predict networks of infiltrated distinct immune-cell phenotypes at higher resolution, we explored an integrated knowledge-based approach to select immune-cell signature genes integrating not only expression enrichment across immune-cells, but also an automatic capture of relevant immune-cell signature genes from the literature. This knowledge-based approach was integrated with resources of immune-cell specific protein networks, to define signature genes of distinct immune-cell phenotypes. We demonstrate the utility of this approach by profiling signatures of distinct immune-cells, and networks of immune-cells, from metastatic melanoma patients who had undergone chemotherapy. The resultant bioinformatics strategy complements immunohistochemistry from these tumors, and predicts both tumor-killing and immunosuppressive networks of distinct immune-cells in responders and non-responders, respectively. The approach is also shown to capture differences in the immune-cell networks of BRAF versus NRAS mutated metastatic melanomas, and the dynamic changes in resistance to targeted kinase inhibitors in MAPK signalling.

**Conclusions:**

This integrative bioinformatics approach demonstrates that capturing the protein network signatures and ratios of distinct immune-cell in the tumor microenvironment maybe an important factor in predicting response to therapy. This may serve as a computational strategy to define network signatures of distinct immune-cells to guide immuno-pathological discovery.

**Electronic supplementary material:**

The online version of this article (doi:10.1186/s12859-016-1141-3) contains supplementary material, which is available to authorized users.

## Background

It is now established through pioneering studies [1, 2], using standard assays and more recently with transcriptomics [2–4], that diverse general types of immune-cells in tumors have differing [5–8] prognostic values across numerous cancer types [7, 9, 10]. Emerging evidence is revealing the importance of a relationship between the clinical response of cancer immunotherapy, and the pre-existing “network” of immune-cells in a tumor’s microenvironment [10–15]. Indeed, it is clear that the adaptive immune response is key in both fighting against cancer progression, and conversely, in shaping an immune resistant microenvironment [5, 14]. Recent success in cancer immunotherapy [16] offer tremendous advance and promise [17]. However, durable responses occur in only a minority of patients. The ability to deduce a more detailed and global “network perspective” of immune-cell functions in the microenvironment [18–20], may improve our understanding of immune resistant phenotypes [21–23]. For example in tumors from non-responders, certain constellations of distinct immune-cells can disarm effective antitumor responses, leading to the emergence of dysfunctional effector cells with diverse immunosuppressive phenotypes. Therefore, it may be of great benefit to automatically and systematically investigate the presence of immune-cell networks in tumors with improved fidelity; i.e. to painstakingly profile a tumor for diverse, precisely defined, distinct immune-cells (such as distinct phenotypes of effector CD8^+^ T cells profiled at high-resolution), rather than general immune-cell types, or generic associations to immune-cells such as “pro-tumor” and “antitumor” [22, 24].

To achieve this efficiently, many systems-immunology challenges need to be overcome; whereby the guidance of improved computational pipelines are needed [21, 25–27]. In this study, we address two such challenges: (a) the attempt to select more precise gene signatures representative of such distinct immune-cell subtypes, and (b) the inference of cooperating networks of these distinct immune-cells in the tumor microenvironment. Here, the term “distinct immune-cells” or DIST, is considered as an immune-cell subtype purified from clearly defined cell surface markers, often characterized by functional status such as naïve, effector, central-memory, and PD1-low, etc. We describe here a novel computational framework that analyzes transcriptomes from tumor biopsies and score them for signatures of distinct immune-cell subtypes (DISTs). This was achieved using a novel bioinformatics framework which defines the gene signatures of DISTs, by (a) using semantic relations of genes to general immune-cell types from the Medline database, computed by a saturation function. Then (b) integrating this information using affinity propagation clustering [28] applied to DIST-specific protein networks. The framework attempts to improve general definitions of immune signatures (such as CD4+ T cells, CD8+ T cells, NK cells, etc.), and offers the possibility to query wide ranges of DISTs in their tumor contexts at multiple stages of differentiation, activation or function.

The selection of immune-cell signature genes for immune profiling in disease has most often relied on differential gene expression [29, 30], enriched expression of transcripts in immune-cells [2, 4, 30], applying expression thresholds across a the immune-cell lineage [31], or identifying modules of co-expressed mRNA transcripts [3, 6]. Similarly, immune-cell specific genes have been identified on the basis of higher gene expression across all immune-cells compared to a selection of non-immune tissues [32]. In general, these approaches can be considered as being primarily centered on the principle that higher expression in an immune-cell type is likely to define a cell’s distinct properties. However this is only one of many features that may be used identify immune genes [32]. Methods relying on enrichment of gene expression have proved useful in identifying signatures of general immune cell types, although it is challenging to pinpoint signatures for highly specific immune-cell subsets, especially for highly similar subsets [33, 34].

Here we propose a knowledge-driven approach, integrated with measures of expression enrichment that attempts to define detailed DIST signature genes. It leverages semantic associations of genes to general cell types, followed by inference of signature genes from DIST-specific protein networks. The strategy is benchmarked against transcriptomes of metastatic melanomas with matching T cell immunohistochemistry as validation. The approach is demonstrated in cases of therapeutic applications, also with metastatic melanomas, whereby patients have been treated with chemotherapy and resistance in combined targeted therapy to MAPK inhibitors. We illustrate the ability of this bioinformatics approach to infer the tumor killing and the immunosuppressive immune-cell networks of therapy response and non-response, respectively (including that of the checkpoint PD1 Low/High CD8+ T cells in tumors prior to administration to chemotherapy).

With such a framework, the cooperative networks of DISTs in tumors and their association to therapy and clinical outcome may be queried systematically and automatically. This bioinformatics approach may serve as a system to help define the molecular signatures of DISTs, which can be used to provide hypotheses to guide immuno-pathological discovery in cancer.

## Results

### A bioinformatics pipeline to identify signature genes from transcriptomes of distinct immune-cells

**Fig. 1:**
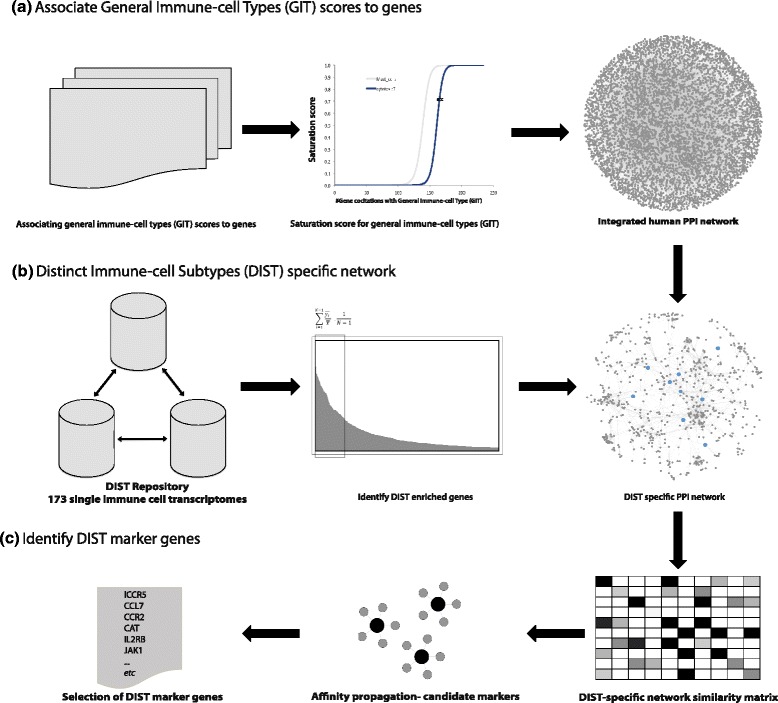
An integrated bioinformatics workflow to capture DIST signatures: **a** Using Medline gene indexes and the gene association to general immune-cell keywords, GIT associated genes are extracted in an automated manner from the literature. **b** Accessing large public resources for DIST transcriptome repositories, DIST enriched genes are determined and used to build DIST-specific protein networks via the integration of the human interactome. **c** Similarity matrices are built using the DIST-specific networks based on similarity in protein interaction partners. The DIST-specific similarity matrices are then subject to affinity propagation, whereby the resulting clusters are used to identify DIST marker genes

First, signature genes associated to general immune-cell types (GIT) were extracted from Medline. In the later steps, these GIT genes are used to generate an extended list of genes signifying an association to their corresponding distinct immune-cell phenotypes (DIST). The complete workflow is depicted in Fig. 1, described in detail in the Methods section, and summarized briefly here. In the first step, the signature genes most representative of the GITs, were captured in an automated manner from approximately 18 million Medline abstracts and mapped to a text-mining index of all official human gene symbols [35]. Genes were then scored for GIT relevance by use of a literature saturation function, which calculated the relevance for a gene to each GIT. The saturation function scores on a range from 0 to 1, and genes were assigned GIT relevance if they had a value greater than 0.9 (Fig. 1a, and Methods section “An automated method to associate general immune-cell types to human genes”).

The next steps in the pipeline were motivated toward identifying DIST genes that correspond to their respective GIT categories. To select the final list of DIST signature genes, the resultant 235 GIT marker genes from the first step outlined in Fig. 1a (see Additional file 1: Table S1) identified using the literature saturation function were used to query protein-protein interaction (PPI) network features from corresponding DIST-specific protein networks (Fig. 1a and b). The protein interactions were derived from an integrated database of 69,809 experimentally verified human PPIs, and this resource was integrated with a repository of DIST gene expression profiles, which was compiled, processed and profiled from datasets queried from the GEO database [36] (Fig. 1b and methods section “Defining immune-cell enriched genes from a comprehensive transcriptome repository of distinct immune-cell subtypes”). Each of the 173 DISTs trancriptomes analyzed (see Additional file 1: Table S1) were independently processed through the bioinformatics workflow in Fig. 1b (to avoid cross platform/study issues). DIST-enriched genes were first identified from each of the transcriptome profiles (Fig. 1b) in order to limit the generation of the resulting PPI networks to DIST relevant genes only. Subsequently, these DIST-enriched genes were used to create 173 separate DIST-specific PPI networks (see Fig. 1b).

The DIST-specific PPI networks and the clustering properties of GIT nodes in these networks, were the foundation upon which the final signature genes for the DISTs were selected for. DIST-specific networks were first generated from the DIST enriched genes (Fig. 1b), for subsequent DIST signature selection through affinity propagation algorithm [28] that was applied to the DIST-specific networks (Fig. 1c). Candidate DIST genes found in the same affinity propagation clusters as GIT genes, and having a degree of literature association to the GIT, were then selected. This resulted in 601 DIST signature genes across the immune-cell transcriptomes. This allowed not only for the opportunity to profile the 173 DISTs, but also that of 8451 DIST combinations (from DIST pairs not of the same general immune type), in tumor transcriptomes, to capture their network signature of immune cells (Fig. 1c and Methods section “Network informed selection score of distinct immune-cell marker genes using affinity propagation”).

The reasoning behind the use of PPI networks as a resource to select DIST signature genes that correspond to their GIT genes is that if both the DIST and their corresponding GIT genes are identified in the same affinity propagation clusters; they may also partake in similar functions, signaling pathways or protein complexes in the immune-cells. Thus, allowing for the selection of potential signature/marker genes for DISTs, from clearly defined immune-cell phenotypes in transcriptome repositories. The source code and required source data to run the workflow describe in Fig. 1 and in the Methods section, is freely available for download at the following location: http://invitro.hpc.uio.no/ImmuneNetScore/.

### Signatures of distinct immune-cell phenotypes correspond with immunohistochemistry findings in metastatic melanomas

**Fig. 2:**
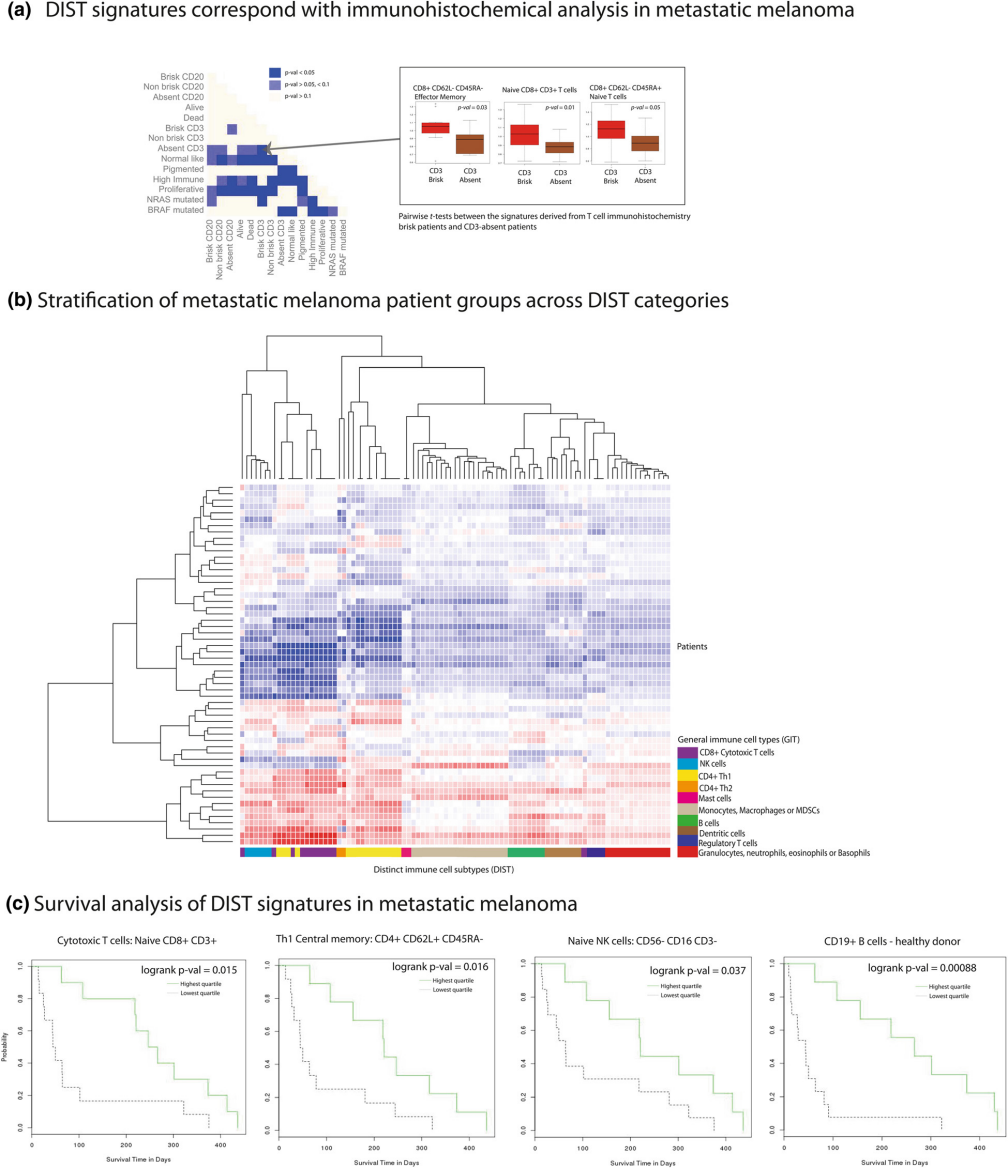
Benchmarking against the immunopathology of the metastatic melanomas: **a** *p* value heatmap and boxplots representing pairwise t –tests for all clinical features and their patient groups. The darkest shade of blue illustrates *p* values < 0.05 (the lighter shade of blue represents *p* values >0.05 and < 0.1). Most notably, for *p* values < 0.05, there was a significant difference between those patient groups positive for a pathological CD3+ brisk infiltrate compared to the patients which were absent for CD3+ brisk. A comparison is labeled dark blue if at least one DIST in the patient group comparison was significant. The three boxplots pointing to that comparison illustrates distinct phenotypes of CD8+ T cells [38, 39], which contribute to the T cell pathology observed in the brisk-positive group. **b** Hierarchical clustering and heatmap (rows represent tumor samples and columns DISTs), across all patients and a comprehensive set of DISTs from several general immune-cell types. The heatmap illustrates clusters of tumor samples that have elevated immune-cell presence in their tumor, and a cluster of patient that are less active for DIST signatures. **c** Kaplan-Meier curves illustrating the three DIST types that correspond to positive outcome in the log-rank difference between the patient groups which were from the highest quartile (*green*), and the lowest quartile (*black*) for the DIST signature score. Patients with the highest quartile signal in their tumors for naïve CD8+ T cells [38], central memory Th1 cells [40], mature inactivated NK cell population of cells [38], and regulatory CD19+ B cells [44] were predictive of positive outcome

In order to establish whether the resulting GIT and DIST signatures from the bioinformatics pipeline described in Fig. 1a correspond to an accurate immune pathology when used to profile a tumor transcriptome, we profiled tumor biopsies where both gene expression was available, and also some validation was available on the same matching samples (e.g., immunohistochemistry). To this end, using melanoma as a test case, we analyzed the transcriptomes of 57 metastatic (Stage III and IV) melanomas [37] (see Fig. 2), which in addition to transcriptomics had matching immunohistochemistry performed for the generic T cell surface marker CD3 (a pathological CD3+ brisk infiltrate). A DIST score for each tumor was determined by calculating the normalized average of the DIST gene expression values across the patient cohort. Using the computational approach, the DIST score for CD8+ positive effective memory T cells [38] and two similar populations of CD8+ Naïve cytotoxic T cells [38, 39] were significantly different between CD3 high and CD3 absent patients (Fig. 2a). When analyzing the complete landscape of all patient tumors across all immune-cell types, a hierarchical clustering corresponding to all the DISTs identified a diverse inter-patient heterogeneity and a clear separation of patients into a dichotomy of immune active and less active tumors (see heatmap Fig. 2b).

To ascertain how the predicted DISTs correspond with survival analysis, we performed logrank tests illustrated in Kaplan-Meier survival curves (see Fig. 2c) for patients that were high (the highest quartile for the DIST score, represented by green lines) and low (the lowest quartile for the DIST score, represented by black lines). From the perspective of benchmarking, focusing on the T cell positive (or a pathological CD3+ brisk infiltrate) signatures specifically, T-helper central memory T cells [40] and naïve CD8+ Cytotoxic T cell [41] signatures signify that our *in silico* method corresponds with the immunohistochemical T cell infiltrate patterns identified with favorable outcome [37]. The logrank *p* value for the naïve CD8+ cytotoxic T cell [41] in Fig. 2c was 0.015, compared to a *p* value of 0.051 when considering the expression of cell surface marker CD8 alone. Likewise, for the CD4+ T-helper central memory T cells in Fig. 2c, the *p* value was 0.016 compared to the highly insignificant value of 0.58 when considering the cell surface marker CD4 alone. This demonstrated not only an effective strategy to pinpoint distinct immune-cell phenotypes at higher resolution possibly infiltrating tumors, but also improved patient stratification for responders and non-responders when considering these DIST features.

In addition to confirming the predictive positive T cell signature with clinical outcome, the analysis identified signatures of immune-cell infiltrates that correspond with the emerging knowledge of melanoma immune pathology. Specifically, we predicted presence of mature naïve Natural Killer (NK) cells [38, 42], which corresponded with improved survival in patients (Fig. 2c), and signatures of adaptive CD19+ B cells [43, 44]. The NK cell gene signatures were not reported from these metastatic melanomas patients previously, but corresponds well with the emerging knowledge of NK cell interactions with melanomas [45]., The most significant DIST signature was that of a predicted B cell infiltrate associated to positive clinical outcome, for a CD19+ B cell phenotype [43], and corresponds with the emerging knowledge of the role of regulatory B cells in the immune pathology of a tumor [44]. A complete list of DISTs that had significant differences (logrank *p* values) in the Kaplan Meier curves is listed in Additional file 2: Table S2, and the contributing marker genes are listed in Additional file 3: Table S3. Of note, as the patients had their biopsies taken with transcriptomes profiled prior to treatment with the chemotherapy doxorubicin; their immune profiles, as illustrated in Fig. 2a and c, are suggestive of a specific adaptive immune response being triggered post-chemotherapy and associated to positive outcome.

### The cooperating network of distinct immune-cells: capturing network-associations to PD1-low CD8+ T cells and positive outcome to chemotherapy

To understand the immune pathology of a tumor more comprehensively, it is important to not only profile single DIST phenotypes and their association to clinical outcome; but also that of the cooperating network of distinct immune-cell populations. In other terms, the “ratio” between different DIST signals, which have a synergistic relationship with each other in the microenvironment, may be a determinant for survival and therapy response. With that in mind, we used this computational procedure to capture the pairwise ratio of each of the DIST scores calculated for each (the DIST score for the tumor was normalized average of the DIST gene expression values across the patient cohort). The DIST score for each tumor was then integrated with the cytokine relationships between the DISTs to infer the possible synergistic network of DISTs in the tumors (seeMethods section “Constructing a network similarity matrix for distinct immune-cell subtypes”).

**Fig. 3:**
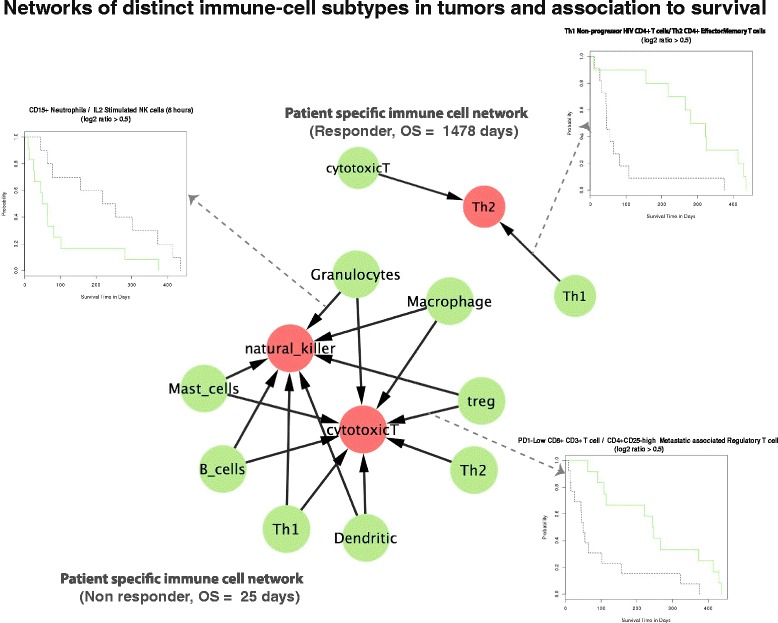
Patient-specific networks of cooperating DISTs in the tumor microenvironment of metastatic melanomas: Edges are included in the network only if the log2 ratio of the DIST pair is greater than 0.5. Nodes are labeled (e.g. Th2) according the corresponding GIT for the DIST being represented. A Kaplan Meier plot for the ratio between the immune cell pair underlies each edge in the networks, but only two Kaplan Meiers are shown for illustration purposes. Green nodes represent the numerator in the log2 ratio calculation and red nodes in the networks represent the denominator, i.e. that the green immune cell type is present in the patient to a greater degree relative to the red immune cell. An example of a responder patient (OS, 1478 days) is shown, top left. The survival analysis for the whole patient cohort (57 patients), illustrated in the figure reveals a classical Th1 driven tumor-killing microenvironment. The highest quartile (*green lines*) and the lowest quartile (*black lines*) of the log2 ratio of the DIST pair are illustrated in the Kaplan-Meier curves. The predicted Th1 cell phenotype [85] is present to a greater extent than Th2 cell phenotype [38] in the network. In addition the responder revealed a greater proportion of cytotoxic CD8+ T cells compared to Th2, revealing a tumor-killing microenvironment network. Conversely, an example of a non-responder patient-specific network (OS, 25 days) is shown (bottom right). Notably, this non-responder hosted a CD8+ cytotoxic T cell subset, low for the checkpoint inhibitor PD-1 [39], to a lesser degree proportional to a T-regulatory cell population DIST [49]. This suggests the presence of a classical immunosuppressive microenvironment in this patient’s tumor. Notably also from the perspective of an immunosuppressive environment is the predicted presence of a higher proportion of a neutrophil DIST population of cells (GSE58173), compared to activated IL2 stimulated NK cells [47]

Connections between a pair of different immune-cells were created if their DIST scores had a log2 ratio greater than 0.5 and the DIST pair had a cytokine receptor interaction with each other. Two such exemplary patient-specific networks applied to the metastatic melanoma patients are illustrated in Fig. 3, in both a responder patient with overall survival (OS) of 1478 days, and a non-responder patient with OS of 25 days, to doxorubicin chemotherapy. The resulting immune-cell networks were suggestive of distinct immune-cell environments being present in the tumor that may determine outcome and also impact the subsequent adaptive immune response to chemotherapy in these patients. In both of the patient-specific immune-cell networks in Fig. 3, the nodes illustrate a GIT associated to one or more corresponding DISTs captured by the immune-cell network analysis. A line connecting two nodes represents a log2 ratio > 0.5 between at least one pair of DISTs. In the case of the responder, a CD+ T-helper 1 cell signature [46] higher than that of the CD4 + T-helper 2 effector memory signature [38] was highly predictive of improved clinical outcome as illustrated by the Kaplan-Meier curves of the responder in Fig. 3. All relationships between DISTs that were significant in the survival analysis for the whole patient cohort are captured in Additional file 4: Table S4 (significant logrank *p* values, for all pairwise log2 ratio scores). The only other network relationship of immune-cell ratios in the same responder patient was a greater CD8+ cytotoxic immune-cell signature compared to the CD4+ T helper cells (Fig. 3). Overall, the responder patient-specific network was suggestive of a classical Th1 driven tumor-killing adaptive immune response with a clear Th1 immune cell phenotype compared to that of a Th2 driven response [38]. In the case of the non-responder, a clear immunosuppressive network of DISTs was inferred by our approach, in addition to other predicted immune-cell ratio relationships (Fig. 3). Two such exemplary DIST relationships are illustrated for the non-responder in Fig. 3. Firstly, it was notable to observe a ratio depicting a higher proportion of CD15+ neutrophils (GSE58173) compared to IL-2 stimulated NK cells [47], which also had a highly significant separation in the Kaplan Meier curves over the whole cohort (Fig. 3). Additionally, as an example of a classically known ratio of an immunosuppressive DIST interaction in melanoma biology [48]; a higher ratio of metastatic associated regulatory T cells [49] compared to CD8+ cytotoxic T cells was observed in the non-responder (Fig. 3). Interestingly, these specific CD8+ T cells were of an effector cell type characterized as “low” for the checkpoint inhibitor PD-1 [39]. Given that CD8+ T cells with high PD-1 expression lose the ability to eliminate cancer due to the checkpoint inhibition, the higher level of signatures of PD-1-low effector CD8+ T cells in patients with positive outcome to chemotherapy is once again suggestive of a downstream adaptive immune response in these patients post chemotherapy administration.

Overall, the summary observations from the patient specific DIST networks in Fig. 3, are indicative of the ability of our approach to capture informative networks of cooperating DISTs for both immunosuppressive and tumor killing responses in metastatic melanoma patients.

### Mutated MAPK signaling and the immunopathology in metastatic melanomas

**Fig. 4:**
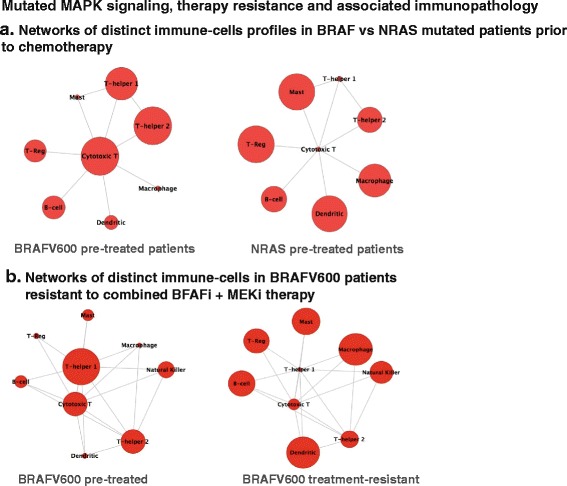
Patient networks (depicting averages over patient groups) of altered immune network landscapes in perturbed MAPK signaling of metastatic melanomas. Nodes are labeled as a GIT which corresponds to at least one DIST. Edges are included in the networks only if the log2 ratio of the DIST pair is greater than 0.5 and their exists a cytokine to cytokine-receptor relationships between the DIST pairs. Both networks in the left and right panels are identical, except for the sides of the nodes, which are scaled proportional to the average GIT immune-cell score for all the patients in the group. **a** As in pre-treated chemotherapy metastatic melanoma patients, there is high CD8 and Th1 immune-cell scores, accompanied by immunosuppressive cells. Possibly indicating a tumor-killing potential pre-therapy with co-occurring immune suppressive barriers. **b** The average size of the immune-cell scores has been visibly altered by the chemotherapy, as visible between pre-treated and resistant patients (naturally, also their respective ratios with each other in the networks). This was most notable for Th1 and CD8+ T cells between both networks

We next investigated the utility of this framework to capture relationships between oncogenic signaling networks in tumor cells, and the corresponding DIST networks in the tumor. The grade III and IV metastatic melanomas under study [37] had also being genotyped for BRAFV600 and NRASV12 mutations, and therefore allowed us to profile for the network of cooperating distinct immune-cells that may differ between the two most often perturbed MAPK signaling systems in melanomas. As depicted in both networks in Fig. 4a, we detected a dichotomy in the inferred immune-network pathology between BRAFV600 and NRAS metastatic melanomas. Each node in the networks in Fig. 4a corresponds to a GIT, which, as in Fig. 3, represents one or more DIST scored in more than one patient (i.e. the average of the patient groups is illustrated if Fig. 4, and this average corresponds to the node size). Each patient group analyzed in both networks was labeled as harboring either a BRAFV600 or NRAS mutation. An edge is connected between to nodes in the network if (a) the log2 ratio between two of their underlying DIST score is greater than 0.5, in more than 10 % of the patients in the entire cohort, and (b) there exists cytokine receptor interaction between the immune-cell pair. The size of the nodes in the BRAFV600 and NRAS network is proportional to the average GIT signature among all the analyzed patient’s samples for the two different mutation types. Most notably, there is a contrasting pattern observed between the CD8+ cytotoxic T cell and Th1 cell responses between BRAFV600 and NRAS melanoma patients. In addition to a dichotomy between the CD8/Treg ratios in comparing both networks, there were altered ratios between macrophages and CD8+ T cells, and between mast cells and CD8+ T cells and Th 1 cells (Fig. 4a). The larger presence of CD8+ cytotoxic T cells and Th1 cells in BRAFV600 metastatic melanomas once again suggests the presence of a potential tumor-killing environment, recruited to the tumor as a result of the BRAF oncogenic insult, but suppressed due to many possible immune-escape mechanisms including that of the high T-helper 2 ratio dominant in the BRAFV600 patient network, as opposed to the low CD8+ and T-helper 1 positive cells in the NRAS patients (Fig. 4a).

In addition, the framework was used to analyze metastatic melanoma patients in a study of perturbed MAPK signaling systems, which have become resistant to combined MAPK inhibitor (MAPKi) drugs [50]. Networks capturing the immunopathology of these patient groups are illustrated for both pre-treated and treatment-resistant BRAFV600-mutant melanomas (see Fig. 4b). These patients consisted of progressing (acquired resistant) and matched pre-treatment melanoma tumor samples [50] treated with combined dabrafenib (BRAF inhibitor) and trametinib (MEK inhibitor). Both of the networks from this MAPK drug resistance study in Fig. 4b were constructed in the same manner as the chemotherapy study in Fig. 4a above: the size of the nodes corresponds to the GIT scores averaged over the patient groups (pre-treated and treatment-resistant). Likewise, there is at least one DIST underlying the GIT labeled nodes, and at least one significant log2 ratio between at least one pair of DIST in the networks.

There are notable differences in the properties of the immune-cell networks between pre-treated and treatment-resistant patient’s tumors. Although averaged over all patients in the group, an altered immune-cell network was detectable between the patient groups. In that regard, there was a difference between CD8+ T cells and Th1 cells, once again indicative of a switch from a “potential” tumor-reactive microenvironment (co-occurring with some immune suppressive factors), to an immune resistant phenotype in the MAPKi treatment resistant patients. In the same regard, differences are visible between ratios of CD8/Treg and CD4/Treg between the two patient groups in Fig. 4b. In this case of targeted MAPKi therapy, it was again suggestive of a transition to an immune suppressive environment in resistant patients with poor outcome, with the therapy resistant network depicting higher signals for diminished ratios of CD8/Treg and Th1/Treg compared to the pretreated cases (Fig. 4b). Additionally, the network analysis reveals interesting trends in an increase of macrophage and dendritic cell signals in treatment resistant patients compared to the pre-treated patients. This is suggestive of an increased immunosuppressive microenvironment contributed by regulatory phenotypes of macrophages and dendritic cells [51] in the MAPKi treatment resistance [52].

## Discussion

We are entering an era where diverse and global immune profiling will routinely guide clinical decision-making and predict response to immunotherapy in disease [53–55]. Transcriptome analysis of distinct immune-cell subsets will have an important contribution toward this effort. As for example, in a recent review, the concept of exploiting the network characteristics of immune cells (based on transcriptional network modules) has been suggested as an important path forward to address the challenges of accurately applying systems immunology in the clinic [21]. Indeed, transcriptional modules have proven to enable systems scale analysis, demonstrating that immune-cell transcriptomics is a powerful source of information on immune-cellular states during health and disease [46, 56, 57]. However, a worthwhile addition to gene expression network analysis to delineate DIST signatures is the immune-cell’s unique spectrum of functional signaling networks [58], and the differential PPI networks between distinct cell subsets [59], as applied in this study. Our exploitation of the unique features of DIST-specific PPI networks may allow for a more precise selection of marker genes representing a DIST that may be used in immune profiling at higher resolution. In principle such a strategy is scalable to identify novel signatures from DISTs, which are isolated, phenotyped and characterized for function from large compendiums of transcriptomes, accumulating in public repositories [36, 38, 60].

One advantage in capturing signals of general immune-cell types from Medline is that we allowed for association to cell types in an automated fashion [61], without reliance on manual curation on the specific categories of immune-cells. In order to achieve a balance of accuracy and automated association to the immune-cell type, there was a strict requirement on the saturation function for genes to have a large amount of co-citations in Medline to be scored positive (which may result in missing some relevant GIT hits for some genes*)*. However, when we map the saturated immune genes to corresponding DIST-specific PPI networks; we allow for an additional layer of capture of DIST-specific marker genes, not dependent on a large coverage in the literature, yet likely to be jointly associated to DIST-specific functions in the cell.

From the perspective of the immune profiling of complex tissue, as in a tumor biopsy, it is a difficult challenge to efficiently and comprehensively capture not only DISTs but also the synergistic network of DISTs in the microenvironment [15]. We have demonstrated that an integrative bioinformatics pipeline can be used to probe systematically and automatically to query for the presence of cooperating DIST networks. Although we will soon have at our disposal powerful technologies to analyze transcriptomes individually at the single cell level [62], it is currently impossible to comprehensively and precisely validate at high-resolution the precise definition of a distinct cell type and its signature/marker genes. However, when analyzed on transcriptomes in metastatic melanomas with accompanying low-resolution validation of immunohistochemistry (IHC) of CD3+ T cells: the approach described here corresponded to IHC findings and recapitulated the role of effector T cells in metastatic melanoma [63, 64]. In particular, this bioinformatics method points toward distinct naïve and memory subsets of CD8 + T cells associated to positive outcome, in addition to immune surveillance by NK cells and regulatory B Cells in tumors of patients with a positive outcome.

Given that biopsies were taken prior to the chemotherapy treatment cycle, such observations allude to the possibility that these tumors undergo an immunogenic cell death through the chemotherapeutic drug (doxorubicin), followed by adaptive immune responses toward exposed antigenic material [65, 66]. This possibility is supported further when we observe T cells low for the PD1 were predictive of positive outcome, and indeed the synergistic (or network) outcome of the PD1-low cells relative to immunosuppressive T regulatory cells revealed a highly predictive response to the chemotherapy: suggestive that the tumor infiltrating CD8+ T cells were not subject to PD1-PDL1 inhibitory signals, in patients with positive outcome, and therefore may have the capacity to mount an immune attack. Interestingly, CD8^+^/Treg ratios have been verified to increase upon combination checkpoint therapy in melanoma, improving survival in melanoma [67], and the CD8^+^/Treg ratio is associated to improved survival in ovarian [68] and gastrointestinal [69] cancers, among other tumor types.

The ratios of diverse DISTs in a tumor and their synergistic effects are important to understand response to therapy. In metastatic melanoma, for example, the cooperation of CD8+ T cells and Natural Killer cells are necessary to mediate anti-tumor activity during combination therapy with IL2 and anti-CTLA [70], and for the use of BRAF inhibitors in metastatic melanoma [71]. Notably, the gene signatures scores for NK and CD8+ immune-cells in this analysis were lower relative to many immunosuppressive cells in the non-responder melanoma patient highlighted in this analysis. The precise network of DISTs in a tumor is likely to be orchestrated by multiple dynamic processes, ranging from its stage in the immunoediting phenotype [72] to the tumor cell type and its perturbed genetic landscape. Therefore, an accurate and comprehensive characterization of the cooperating DIST network in tumors is very difficult. This computational approach may lessen this difficulty and may serve as a guide to expedite the discovery process of the precise immune-cell landscape in a tumor.

From the point of view of melanoma and targeted therapy, via inhibitors of the MAPK pathway, such profiling of the networked landscape of immune-cells may be of great importance, due to the burgeoning evidence of the role of immune modulation in MAPKi therapy [73–75]. The computational approach here detected notable differences in the immune-cell networks between BRAF and NRAS mutated metastatic melanomas, in addition to BRAF mutated melanomas that have acquired resistance after combined MAPKi therapy. The approach illustrated that, on average, pretreatment tumors harboring a BRAF mutation have higher signals for distinct CD8+ T cells and Th1 cell signatures, signifying a tumor-killing environment. Given that BRAF and NRAS mutated tumors, lacking immune-related gene expression signatures, have poor outcome [76]; it is may be of value to apply this computational query system to systematically probe and score for the possible immune landscape in tumors before the administration of therapy.

It is important to consider that the density, distribution and precise location of distinct immune cells within the tumor are factors not analyzed by this this computational approach. These are important prognostic and diagnostic elements not considered by our approach. However, these are features of critical importance, as indeed evidenced in melanoma [77, 78] and as first highlighted in colorectal cancer [1]. To accurately profile these spatial and temporal dynamics, we must continue to rely on classical immune profiling methods (IHC, flow cytometry, etc.), especially those which apply a systems-biology perspective [2], and also integrate novel methods in the emerging field of single-cell technologies [79]. In the near future we will have the opportunity to analyze a plethora of DISTs profiled at the single-cell level. However, to interpret such data, bioinformatics strategies to help define signatures genes of such DISTs as attempted here may be necessary. This is particularly the case when considering the advent of improved methods to algorithmically deconvolute the quantity of rare cellular populations from the transcriptomes of complex tissue [33, 34].

## Conclusions

Many previously reported and emerging deconvolution algorithms require precisely defined marker genes or expression profiles prior to implementation [33]. Therefore, new integrative bioinformatics approaches such that as proposed here may be useful to define signatures marker genes. This may especially be the case for small yet highly similar functionally important populations of DISTs profiled at the resolution of the single-cell. In particular for cancer, strategies that incorporate such methods, may guide us in defining the network of functionally important DISTs to discover predictive biomarkers for therapeutic response, and support the discovery of mechanisms that undermine the interplay between a tumor and the network of immune cells in its microenvironment.

## Methods

### An automated method to associate general immune-cell types to human genes

The general immune-cell types (GIT) we chose to analyze were macrophages, dendritic cells, CD8+ T cells, CD+ Th1 cells, CD4+ Th2 cells, CD4+ regulatory T cells, natural killer cells, B cells granulocytes (both eosinophils and neutrophils), myeloid derived suppressor cells (MDSCs), and mast cells. For each GIT, the most relevant Medical Subject Headings (MeSH) term-codes representing that general cell type were selected manually. The index of these MeSH terms to Medline abstracts from the National Library of Medicine’s (NLM) annotations of MeSH terms to articles was then retrieved. Next, all official gene symbols from the Human Genome Organization (HUGO) were retrieved from a text-mining index of Medline abstracts using a natural language processing (NLP) database [35]. The MeSH term-code index and this gene text-mining index were then cross-referenced and their co-citations in Medline enumerated. For each gene *i*, a general immune-cell type relevance score *S*_3_, which ranges between *S*_3_, (0) = 0 and *S*_3_, (∞) = 1, was calculated for each GIT using the following saturation function:

$$ {S}_3=\frac{1}{1+x,{e}^{-y}}x={a}^2,y=\frac{b^2}{a} $$

This relevance score *S*_3_ was modeled as a non-linear saturation curve (logistic function) where the constant *a* is the total number of co-citations among the immune-cell type’s MeSH term-codes in Medline, and the variable *b* is the total number of co-citations with these MeSH terms for gene *i*. The behavior of the function above that calculates the relevance score was designed such that *y* controls for a strict degree of steepness of the logistic curve. Thus, the saturation score behaves such that the greater the degree of co-citation of a gene with the relevant immune-cell MeSH terms in Medline; the increased likelihood there is that the gene will reach the steep saturation point set to ensure the immune-cell relevance. In other terms, for a gene to reach saturation point, it is necessary to have a large number of co-citations with the immune cell’s MeSH terms relative to the total number of citations of the MeSH terms. Because of the necessity in having a relatively high co-citation value, this relevance score *S*_3_ as calculated represents an automated association of gene *i* to the general immune-cell types, while simultaneously correcting for possible literature bias through demanding a steep saturation curve for relevance in the biomedical literature (see saturation curves, demonstrated for cytotoxic T cells and Mast cells in Fig 1a).

A very small number of well-known signature genes of immune-cells were reserved for association to specific GIT categories. If any of these genes were captured by the saturation function for their corresponding well-known GIT, they were then reserved for that cell type, and therefore not considered if captured by the literature saturation function for the other GITs. This step addressed the inherent noise in the text mining indexes of Medline abstracts, while also allowing the saturation function to score most human genes to general immune-cells types, in an automated manner.

### Defining immune-cell enriched genes from a comprehensive transcriptome repository of distinct immune-cell subtypes

A large and detailed repository of Distinct Immune-cell Subtypes (DIST) transcriptome datasets was compiled from the Gene Expression Omnibus (GEO) database [36]. The DIST repository consisted of 551 transcriptomes from a total of 173 distinct profiles of immune-cells, characterized in 28 human single-cell datasets (Additional file 1: Table S1). Each of the datasets involved the purification of the single-cells from the immune system, followed by gene expression profiling. The set of immune-cell enriched genes for each DIST where determined separately for each transcriptome dataset processed in the repository. Firstly, each individual dataset was separately normalized using quantile normalization [80]. Then, the mean value $\overline{x}$, for the target DIST was calculated among its replicates. Subsequently, the ratio of *y* to the mean value for each remaining DISTs in the dataset was calculated, summed and averaged as follows:

$$ {\displaystyle \sum_{i=1}^{N-1}\frac{\overline{y_i}}{\overline{x}}}\cdot \frac{1}{N-1} $$

Where, for example, $\overline{y_i}$ is the mean of one of the remaining DIST profiles *i* ∈ (1 → *N*), and *N* is the total number of distinct single immune-cells in the dataset. The resulting gene values were then sorted in ascending rank-order with a lower value representing higher expression in the target DIST. The top 10 % ranked genes were assumed as being enriched for the target DIST, and later selected for in the protein network informed procedures to identify it’s DIST marker genes (see Methods below). The DIST enriched genes were defined separately in this manner on a per dataset basis across the repository (see Additional file 1: Table S1) in order avoid the many possible error prone complexities of dealing with cross- platform, −laboratory and -experimental sources of bias between the different studies in the DIST transcriptome repository.

### Construction of distinct immune-cell specific networks

Networks of the protein products of human genes were sourced from 10 integrated protein-protein interaction (PPI) databases, structured by the iRefIndex [81]. The PPI databases were downloaded from the binary and physical protein associations through the iRefWeb service [82, 83]. The interactions are integrated in this resource by mapping identifiers across the databases with systematic backtracking to establish the non-redundant identity of interacting partners. A strict filtering process for each PPI was applied, whereby only physical binary protein interactions that satisfied all of the following criteria were selected: (*a*) experimentally verified; (*b*) both interactors originate in human; (*c*) at least one supporting publication in Medline, and (*d*) physically binding PPIs. For each of the DIST profiles, their immune-cell enriched genes (a strict threshold of the top 10 % ranked genes, described above), were mapped to the integrated PPI network to create their DIST-networks. These top 10 % single-cell enriched genes in a DIST were then used as seed genes to query the integrated PPI network and connections were only allowed to be formed in the resulting network if an interaction partner was among a certain top percentage enriched genes (30 % in these studies profiled here, but configurable by the pipeline); creating DIST-specific networks across the whole dataset, and subsequently the DIST repository.

### Constructing a network similarity matrix for distinct immune-cell subtypes

For each DIST-specific network, a similarity matrix was built by calculating a similarity metric for all protein pairs in the network. For a protein pair A and B, shared interactions between the pair in the DIST-network were calculated as (|*N*(*Λ*) ∩ *N*(*B*)|) in relation to the connectivity, or degree centrality, of *N*(*Λ*) and *N*(*B*) in the entire DIST-specific network. The Simpson index was then used as the similarity metric, calculated as the proportion of shared interactions between the protein pair relative to the degree of the least-connected protein of the pair in the network.

$$ \frac{\left|N\left(\varLambda \right)\cap N(B)\right|}{ \min \left(\left|N\left(\varLambda \right)\right|,\left|N(B)\right|\right)} $$

For each of these similarity metrics, a real-valued matrix S was then formulated, in which a pairwise comparison *S*_*AB*_, corresponded to a value representative of the similarity of protein *A* to protein *B* in the DIST-specific network.

### Network informed selection score of distinct immune-cell marker genes using affinity propagation

The goal of this component was to expand the GIT marker genes identified above and use the molecular network of single-cells (the DIST-specific networks) to inform the selection of DIST-specific marker genes. To that end, the real valued similarity matrices (described above) were used as input into a specialized clustering algorithm, Affinity propagation (AP), to identify exemplifying clusters [28] of genes holding similar interaction partners in the DIST-specific network. Briefly explained, the AP clustering algorithm operates by passing bi-directional messages of similarity values between all pairs of data points in the similarity matrix until a set of exemplifying clusters emerge as the algorithm iterates. For a gene pair A and B, there are two different types of messages exchanged, resulting in two different matrices where operations are carried out: the “availabilities” matrix (*a*(*Λ*, *B*)) and “responsibilities” matrix (*r*(*Λ*, *B*)). The “availability”, sent from candidate exemplar gene B to A, is a query of how gene B is suitable to be available for gene A to become an exemplar cluster. The “responsibility”, sent from gene A to gene B, is a query of how gene A in the message passaging system is suitable to serve as exemplar in B. The values in both matrices are computed as log likelihood ratios and the availability is fixed initially at zero. The responsibility matrix is then updated while the availability matrix updates and accumulates scores from all possible genes as to their likelihood of being optimal exemplar clusters (computed as the sum of the responsibilities (*r*(*Λ*, *B*)). More extensive details of the affinity propagation algorithm, its update functions, and its approximations are available by Frey and Dueck [28], describing its source development. The affinity propagation clustering algorithm holds the advantage over classical clustering procedures to identify DIST-specific markers in that it considers, simultaneously and unbiased, all proteins in the DIST-specific network as potential members of the exemplifying cluster. Additionally, the algorithm generates deterministic results, and robust DIST-specific markers can be efficiently produced for each analysis. The overall DIST score for the resultant signature marker genes, and their pairwise ratio scores, for each DIST identified using this approach, are profiled in a tumor transcriptome mixture by the normalized average across the patient cohort in the analyzed metastatic melanoma study (see below).

### Constructing a template and tumor-specific networks of distinct immune-cell interactions

The cytokine relationships between two DISTs were constructed in a similar manner to the immune body cytokine network [84], whereby the soluble factors which link immune cells together are integrated. Cytokine-receptor interactions were complied from two main databases: the Cytokines Online Pathfinder Encyclopedia (COPE) and the Cytokine Reference Online Database, as reported previously [84]. Based on these cytokine-receptor relationships between immune-cells, a template immune cell network was formed. In this template network, immune-cells were represented as nodes and cytokine interactions between any pair of immune-cells were represented as edges. The template network consisted of the nodes of each GIT and their 29 possible edges (whereby each edge represented one of the main cytokine interactions among immune cells). The network construction, based on a specific tumor biopsy analysis, allowed cytokines to connect two or more cells if one of the cells is known to release cytokines to which the other responds through a cytokine-receptor interaction (i.e., present in the template network). Additionally, edges were pruned from this template immune cell network if the log2 ratio of at least of one pair of DIST underlying their corresponding GIT nodes was less than a conservative value of 0.5.

## Abbreviations

AP, affinity propagation; DIST, distinct immune-cell subtype; GIT, general immune cell type; IHC, immunohistochemistry; MeSH, Medical Subject Headings; NLP, natural language processing; PPI, protein-protein interactions network

## Additional files

Additional file 1: Table S1. List of distinct immune-cell subtypes (DIST) used consisting of 551 transcriptomes from a total of 173 distinct profiles of immune-cells, characterized in 28 human single-cell datasets with PubMed IDs. (XLS 90 kb) Additional file 2: Table S2. List of DISTs with significant differences (logrank *p* values) in the Kaplan Meier curves between groups in highest and lowest quartile for the DIST, in a metastatic melanoma cohort of patients who have undergone chemotherapy. (XLS 30 kb) Additional file 3: Table S3. The resultant general immune-cell type (GIT) and DIST marker genes identified using the bioinformatics workflow. (XLS 85 kb) Additional file 4: Table S4. List of DISTs ratios with significant differences (logrank *p* values) in the Kaplan Meier curves between groups in highest and lowest quartile for the DIST ratio, in the metastatic melanoma cohort of patients. (XLS 215 kb)
